# Identification of potential ferroptosis hub genes in acute-on-chronic liver failure based on bioinformatics analysis and experimental verification

**DOI:** 10.1186/s12920-023-01480-4

**Published:** 2023-03-11

**Authors:** Meixia Kuang, Longhui Cai, Jing Zhao, Liqiao Huang, Yichun Ji, Bingyao Lv, Weihong Kuang

**Affiliations:** 1 1St School of Medicine, Guangzhou University of Chinese Medicine, Guangzhou, 510405 China; 2 School of Pharmacy, Guangdong Medical University, Dongguan, 524023 China; 3 Shenzhen Bao’an Traditional Chinese Medicine Hospital, Guangzhou University of Chinese Medicine, Shenzhen, 518133 China; 4 Guangdong Key Laboratory for Research and Development of Natural Drugs, School of Pharmacy, Guangdong Medical University, Dongguan, 524023 China

**Keywords:** Acute-on-chronic liver failure (ACLF), Ferroptosis, Bioinformatics analysis, Gene

## Abstract

**Background:**

Ferroptosis plays an important role in the development of acute-on-chronic liver failure (ACLF). The present project aimed to identify and validate the potential ferroptosis-related genes in ACLF by bioinformatics analysis and experimental verification.

**Materials and methods:**

The GSE139602 dataset was obtained from the Gene Expression Omnibus database and intersected with ferroptosis genes. Ferroptosis-related differentially expressed genes (DEGs) between the ACLF tissue and healthy group were analyzed using bioinformatics methods. Analysis of enrichment, protein‒protein interactions, and hub genes was conducted. Potential drugs targeting these hub genes were retrieved from the DrugBank database. Finally, we performed real-time quantitative PCR (RT-qPCR) to validate the expression of the hub genes.

**Results:**

A total of 35 ferroptosis-related DEGs were screened, which were enriched in the biosynthesis of amino acids, peroxisomes, fluid shear stress and atherosclerosis. PPI network analysis indicated five ferroptosis-related hub genes, namely, HRAS, TXNRD1, NQO1, PSAT1, and SQSTM1. The experimental validation indicated that the expression levels of HRAS, TXNRD1, NQO1, and SQSTM1 were lower, while the expression level of PSAT1 was higher in ACLF model rats than in healthy rats.

**Conclusions:**

Our findings reveal that PSAT1, TXNRD1, HRAS, SQSTM1 and NQO1 may affect the development of ACLF by regulating ferroptotic events. These results provide a valid reference for potential mechanisms and identification in ACLF.

**Supplementary Information:**

The online version contains supplementary material available at 10.1186/s12920-023-01480-4.

## Background

Acute-on-chronic liver failure (ACLF) is an acutely exacerbated chronic liver disease, accompanied by the rapid progression of multiple organ failure and high short-term mortality. With the expansion of liver disease and hepatocirrhosis worldwide, the incidence of ACLF is rising yearly. However, effective medical treatment has yet to be discovered. Obviously, effective treatment for the disease has become an important medical issue.

**Fig. 1:**
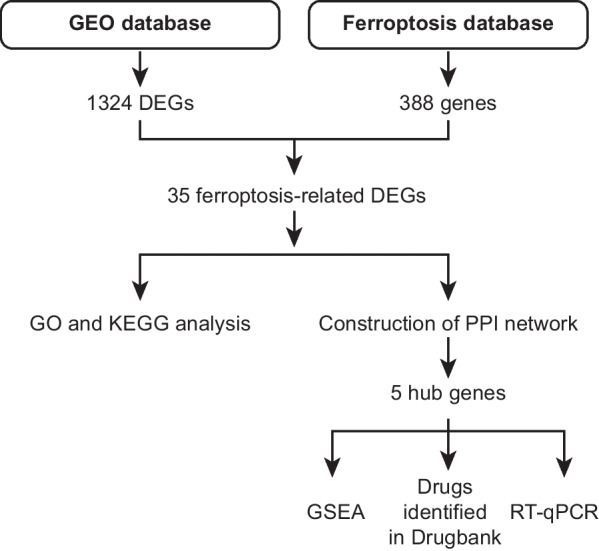
Flow chart of the study. GEO: Gene Expression Omnibus; DEGs: Differentially expressed genes; GO: Gene Ontology; KEGG: Kyoto Encyclopedia of Genes and Genomes; KEGG: Kyoto Encyclopedia of Genes and Genomes; GSEA: Gene Set Enrichment Analysis; RT‒qPCR: Real-time quantitative PCR

Effectively preventing liver cell death in patients with ACLF may be the key to treatment. Recently, a novel type of cell death has been discovered—ferroptosis, which is characterized by iron ion accumulation in the cell, imbalanced redox reactions, and enhanced lipid peroxidation, eventually leading to cell membrane rupture and cell death [1]. These characteristics were also observed in a hepatic failure model [2]. Studies have revealed that liver diseases are caused in part by high levels of iron and lipid accumulation from reactive oxygen species (ROS) [3, 4]. It has been found that acetaminophen (APAP) can cause liver lipid peroxidation and ferroptosis, which leads to acute liver failure (ALF) in the mice, while treatment with antioxidants (liproxstatin-1, vitamin E, ferrostatin-1) or iron chelators (deferoxamine) can reverse the lipid peroxidation of ferroptosis and treat ALF [5]. From what has been discussed above, ferroptosis can affect liver disease by regulating the process of iron ions at the cellular level and the degree of lipid peroxidation; thus, liver failure can be effectively prevented and treated by inhibiting ferroptosis of liver cells [6]. Thus, it is important to identify the precise molecular mechanisms involved in ferroptosis in ACLF and to devise effective diagnostic and therapeutic methods. In this study, we used bioinformatics to screen ferroptosis-related differentially expressed genes (DEGs) in ACLF. Then, we performed real-time quantitative PCR (RT‒qPCR) to validate these hub genes. Figure 1 shows the flow chart of the study. Our study provides insights into the mechanism of ferroptosis in ACLF, discusses novel methods to clinically diagnose and treat ACLF and suggests corresponding targets for subsequent systematic research.

## Materials and methods

### Screening of microarray data

Patients with ACLF were obtained from the NCBI Gene Expression Omnibus (GEO: https://www.ncbi.nlm.nih.gov/). We downloaded the GSE139602 dataset derived on the GPL13667 platform (Affymetrix Human Genome U219 Array). In this database, transcriptome analysis of liver biopsies from patients at different liver disease stages, including 5 fibrosis patients, 8 compensated cirrhosis patients, 12 decompensated cirrhosis patients, 8 ACLF patients, and 6 control healthy individuals, was performed [7]. The data from the 8 ACLF patients and 6 control healthy individuals were selected for further analysis.

### Identification of DEGs related to ferroptosis

The GSE139602 dataset was first normalized using the normalized between arrays function in the R software limma package [8] to avoid a high false discovery rate (FDR), and p values were also estimated for FDR control. Next, the DEGs between patients and normal controls in the dataset were screened. DEG identification threshold values were set at | log2-fold change (FC) |≥ 1 and* P* < 0.05. Then, we downloaded a dataset that included 388 genes from the Ferroptosis Database (FerrDb) [9] (http://www.datjar.com:40013/bt2104/) and compared it with the GSE139602 dataset to screen for ferroptosis-related DEGs. Using Venny2.1 (https://bioinfogp.cnb.csic.es/tools/venny/index.html), we created a Venn diagram of the DEGs. The R software “ggplots” and “pheatmap” packages were used to plot the volcano map and heatmap, respectively.

### Functional enrichment analysis

Functional enrichment analysis of DEGs was conducted with WebGestalt (http://www.webgestalt.org/), DAVID 6.8 (https://david.ncifcrf.gov), and the ClusterProfiler package in R. We uploaded the ferroptosis-related DEGs to the Gene Set Enrichment Analysis (GSEA) tool in WebGestalt for further analysis. Initially, GSEA filtered the gene sets according to the number of genes contained, with a minimum number of three genes and a maximum number of 2,000 genes per gene set. The Kyoto Encyclopedia of Genes and Genomes (KEGG) [10] analysis was obtained from GSEA. DAVID 6.8 was also used for KEGG enrichment analysis to identify the potential pathways that the target genes participate in. Moreover, we carried out Gene Ontology (GO) and KEGG enrichment analyses of ferroptosis-related DEGs using the enrichKEGG and enrichGO functions of the ClusterProfiler package in Bioconductor (http://bioconductor.org/packages/release/bioc/html/clusterProfer.html) [11]. *P* < 0.1 and count ≥ 2 were chosen as the cutoff values for identifying ferroptosis-related DEGs in terms of biological processes (BPs), cellular components (CCs), and molecular functions (MFs). After that, we carried out KEGG pathway enrichment analysis using *P* < 0.2 and count ≥ 2 as cutoff values. Gene set enrichment analysis (GSEA) was also performed using the ClusterProfiler package to investigate the biological functions of hub genes. Samples were separated into two groups based on the median values of the hub gene expression group. Genes were sorted by logFC from the highest to the lowest, and the background gene set was downloaded from MSigDB [12]. To show the results of enrichment analysis, the barplot, dotplot and enrichplot packages were used.

### Construction of the PPI network and identification of hub genes

A protein‒protein interaction (PPI) network of ferroptosis-related DEGs was constructed using STRING (version 11.5, https://cn.string-db.org/) [13]. The visualization was performed using Cytoscape (version 3.8.2, https://cytoscape.org/) with a confidence score > 0.15 [14]. We used Molecular Complex Detection (MCODE) in Cytoscape for cluster analysis in the PPI network with a degree cutoff = 2, node score cutoff = 0.2, kcore = 2, and max depth = 100 [15]. The modules with established scores > 5 were selected. In addition, CytoHubba in Cytoscape was used to discover the top 10 node genes in 10 ways and set the intersection to filter the hub genes. The most accurate (MCC) method was used in CytoHubba [16]. Then, we selected the downregulated hub gene with the highest MCC score, HRAS, as the key gene and predicted the first gene that interacted with it by using CytoHubba.

### Drugs identified in DrugBank

Drugs targeting the identified hub genes were retrieved from the DrugBank database [17]. The DrugBank database is a cheminformatics and bioinformatics repository containing detailed information on drugs and their targets, with information on more than 7,800 drugs.

### Experimental animals and ACLF models

Adult male Sprague‒Dawley rats were purchased from Beijing HFK Bioscience Co., Ltd. According to protocols approved by the Animal Care and Use Committee at the Beijing HFK Bioscience CO., LTD Peking University Shenzhen Graduate School (Shenzhen, China), all experimental rats were provided free access to water and food. This experiment was approved by the Animal Ethics Committee of Shenzhen Peking University Hong Kong University of Science and Technology Medical Center, China (No:2022-194), and all methods were carried out in accordance with relevant guidelines and regulations. This study was carried out in compliance with the Animal Research: Reporting of In Vivo Experiments (ARRIVE) guidelines. The rats were separated into two groups (the control group and ACLF model group) with 6 rats in each group. With reference to Cong W [18], rats were anesthetized with 5% isoflurane (#R510-22-10, Shenzhen Redwood Electronic Technology Development Co., Ltd.) and remained anesthetized with 4% isoflurane during the procedure. Rats were given a bile duct ligation operation, and a rat biliary cirrhosis occurs 1 month later. The rats were given equal doses of saline. Intraperitoneal injection of 600 mg/kg D-galactosamine (D-GalN) and 20 μg/kg lipopolysaccharide (LPS) was performed for the model group for 24 h on the fourth day. Liver tissues (Additional file 1: Fig. S1) were extracted for further investigations. The same liver tissue was collected from each rat. After washing with phosphate-buffered saline solution, the tissues were fixed in 10% formalin solution for 12–24 h, followed by paraffin embedding, sectioning, and hematoxylin–eosin (HE) staining. Finally, microscopy was used to observe the structural and pathological changes in liver tissue in each group.

### RT‒qPCR analysis

Rat liver tissues were lysed with RNA lysis buffer and ground with a high-speed tissue grinder (Tiss-24, Shanghai, China). All RNA extraction was performed according to the instructions of the RNA extraction kit (Promega, WI, USA), and the concentration and purity of RNA were tested by a NanoDrop-2000 spectrophotometer (Thermo Fisher Scientific, Waltham, MA, USA). Then, the RNA was reverse transcribed into cDNA using Evo M-MLV RT Premix for qPCR (AG11706, Accurate Biotechnology, HUNAN, China). Finally, RT‒qPCR experiments were performed using a SYBR Green Premix Pro Taq HS qPCR Kit (AG11701, ACCURATE BIOTECHNOLOGY, HUNAN, China). The primers for HRAS, NQO1, TXNRD1, PSAT1, SQSTM1, and beta-actin were supplied by Tsingke Biotechnology Co., Ltd. (Beijing, China). Information on the primers is listed in Table 1.

**Table 1:** Specific primer sequences used in RT‒qPCR

Gene name	Primer sequences	
β-actin	FORWARD	TTCGCCATGGATGACGATATC
	REVERSE	TAGGAGTCCTTCTGACCCATAC
SQSTM1	FORWARD	CCAGCACAGGCACAGAAGATAAGAG
	REVERSE	TCCCACCGATCCAAGGCTATC
PSAT1	FORWARD	TCGCTGGTGCTCAGAAGAATGTTG
	REVERSE	TCAAGGACTGATGGCACTCTCTG
HRAS	FORWARD	GCCATCAACAACACCAAGTCCTTTG
	REVERSE	GCACCATTGGCACATCATCTGAATC
NQO1	FORWARD	AGGATGGGAGGTGGTCGAATCTG
	REVERSE	GCCTTCCTTATACGCCAGAGATGAC
TXNRD1	FORWARD	CACGGATGAGGAGCAGACCAATG
	REVERSE	CATACAGCCTCTGAGCCAGCAATC

### Statistical analysis

Both the Statistical analysis and the charts were generated and drawn with GraphPad Prism 8.0 (GraphPad Software, San Diego, CA), respectively. Data are expressed as the mean ± standard deviation. Student’s t test was used to compare the significant difference between the means of the two groups, and the analysis results were considered to be statistically significant when *P < 0.05, **P < 0.01, or ***P < 0.001.

## Results

### Ferroptosis-related DEGs in ACLF

**Fig. 2:**
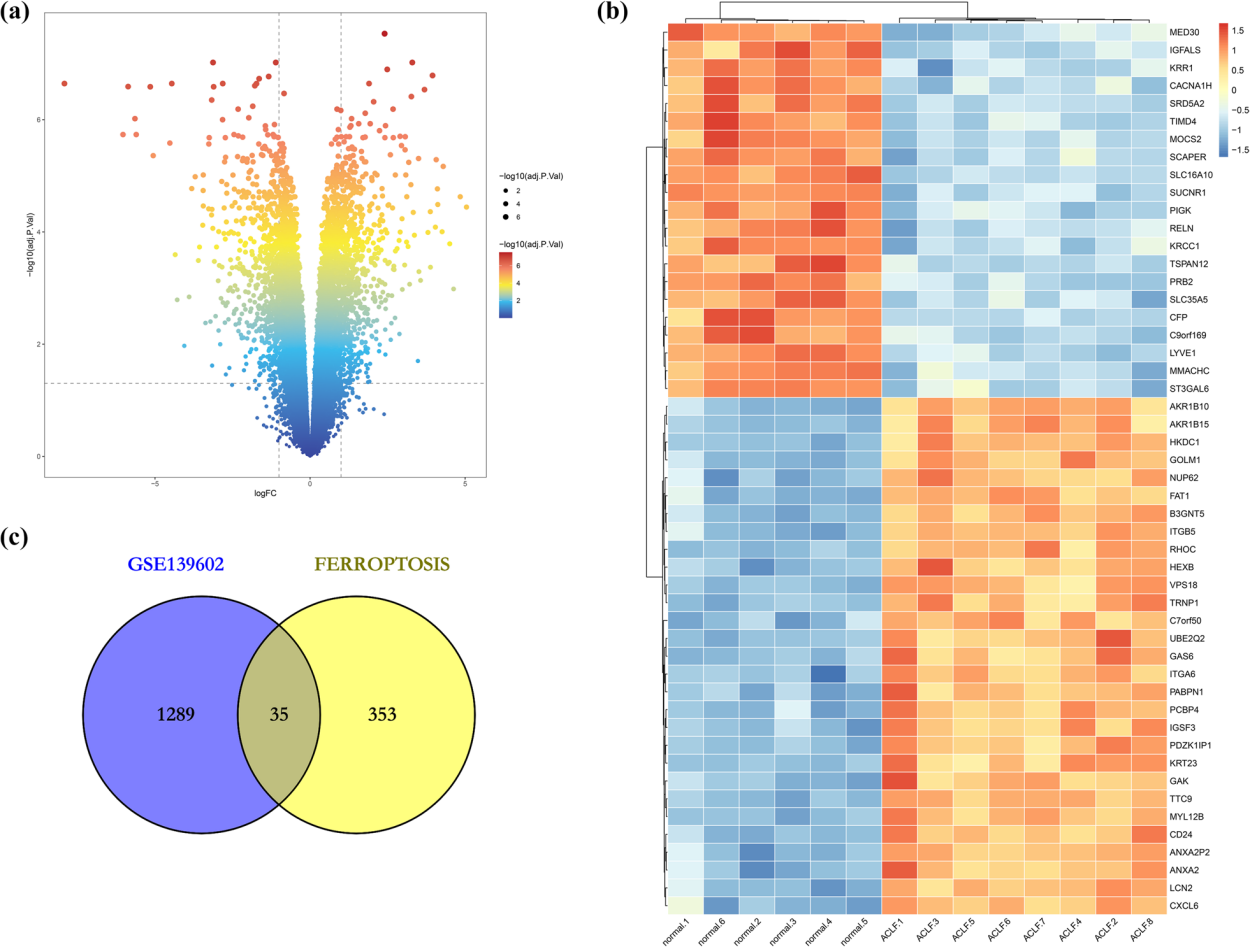
**A** Volcano map of DEGs in the ACLF group and control group. Two vertical lines indicate gene expression fold change (FC) > 2 and < − 2, respectively, and the horizontal line indicates the adjusted *P* value (FDR q-value) of 0.05. *P* values were calculated by two-sided Wilcoxon rank-sum test. The color of the dot represents the FDR (q-value) levels. **B** The first 50 DEGs are shown in the heatmap, with red representing significantly upregulated genes and blue representing significantly downregulated genes in the samples. **C** Venn diagram of ferroptosis-related DEGS. We intersected the ferroptosis dataset with GSE139602 to identify ferroptosis-related DEGs. Red indicates upregulation, and blue indicates downregulation

The GSE139602 dataset was obtained from the GEO database, which included 8 ACLF and 6 control healthy livers. We found 699 upregulated DEGs and 625 downregulated DEGs in the GSE139602 dataset (Fig. 2A). We also obtained a dataset of 388 genes from FerrDb and then compared them with GES139602 to identify ferroptosis-related DEGs. Accordingly, 19 upregulated and 16 downregulated genes were found (Table 2). Figure 2B, C show the heatmap and Venn diagram of the DEGs. The DEGs were further categorized as driver, suppressor, and marker genes by the FerrDb online tool (Table 3).

**Table 2:** Ferroptosis-related differentially expressed genes in ACLF

Gene symbol	*P* Value	logFC	Gene title
*Upregulated genes*			
NR5A2	1.07E−07	1.28702859	NUCLEAR receptor subfamily 5 group A member 2
PANX1	1.55E−07	1.24766675	pannexin 1
ARRDC3	1.13E−06	2.31511952	Arrestin domain containing 3
PSAT1	1.43E−06	2.96649694	Phosphoserine aminotransferase 1
TIMM9	1.77E−06	1.00707478	Translocase of inner mitochondrial membrane 9
PEX3	3.49E−06	1.32833114	Peroxisomal biogenesis factor 3
CDO1	4.25E−06	1.14600079	Cysteine dioxygenase type 1
ACADSB	5.42E−06	2.22836882	Acyl-CoA dehydrogenase short/branched chain
SESN2	8.40E−06	1.16331947	Sestrin 2
AR	4.44E−05	1.0473481	Androgen receptor
SLC2A12	6.52E−05	1.45404063	Solute carrier family 2 member 12
TTPA	0.0001528	2.08107407	Alpha tocopherol transfer protein
PEX12	0.00018338	1.04713249	Peroxisomal biogenesis factor 12
SLC16A1	0.00029344	1.6640174	Solute carrier family 16 member 1
DUSP1	0.00035585	1.80166982	Dual specificity phosphatase 1
TUBE1	0.00797734	1.00822644	Tubulin epsilon 1
DDIT4	0.01616721	1.1277138	DNA damage inducible transcript 4
HAMP	0.01668347	1.86930843	Hepcidin antimicrobial peptide
DPP4	0.01743001	1.18827786	Dipeptidyl peptidase 4
Downregulated genes			
ACSL4	5.93E−08	−1.9087248	Acyl-CoA synthetase long chain family member 4
BCAT2	7.20E−07	−1.6961856	Branched chain amino acid transaminase 2
HRAS	1.04E−06	−1.0135424	HRas proto-oncogene, GTPase
NQO1	1.15E−06	−3.1751323	NAD(P)H quinone dehydrogenase 1
DAZAP1	1.54E−06	−1.3918441	DAZ associated protein 1
HSPB1	4.39E−06	−1.3147566	HEAT shock protein family B (small) member 1
SLC38A1	4.72E−06	−1.3763065	Solute carrier family 38 member 1
SQSTM1	1.10E−05	−1.8764274	Sequestosome 1
SETD1B	4.16E−05	−1.0151086	SET domain containing 1B, histone lysine methyltransferase
ASNS	7.79E−05	−1.691081	Asparagine synthetase (glutamine-hydrolyzing)
CAPG	8.22E−05	−1.7470416	Capping actin protein, gelsolin like
CDKN1A	0.00035835	−1.9259144	Cyclin dependent kinase inhibitor 1A
GPX2	0.0006198	−3.2113914	Glutathione peroxidase 2
NEDD4L	0.00076102	−1.0016884	NEDD4 like E3 ubiquitin protein ligase
TXNRD1	0.00243742	−1.1141324	thioredoxin reductase 1
GDF15	0.00416958	−2.0776601	GROWTH differentiation factor 15

**Table 3:** The differentially expressed ferroptosis genes were divided into ferroptosis drivers, suppressors, and markers

Driver	Suppressor	Marker
ACSL4, HRAS, SLC38A1, DPP4, CDO1, PANX1, PEX12, TTPA, TIMM9, PEX3, ACADSB	NR5A2, BCAT2, NQO1, DAZAP1, SQSTM1, AR, SLC16A1, CDKN1A, NEDD4L, HSPB1, SESN2, GDF15	DUSP1, TXNRD1, GPX2, DDIT4, ASNS, PSAT1, TUBE1, ARRDC3, SETD1B, HAMP, SLC2A12, CAPG, HSPB1, SESN2, GDF15

### Results of enrichment analysis

**Fig. 3:**
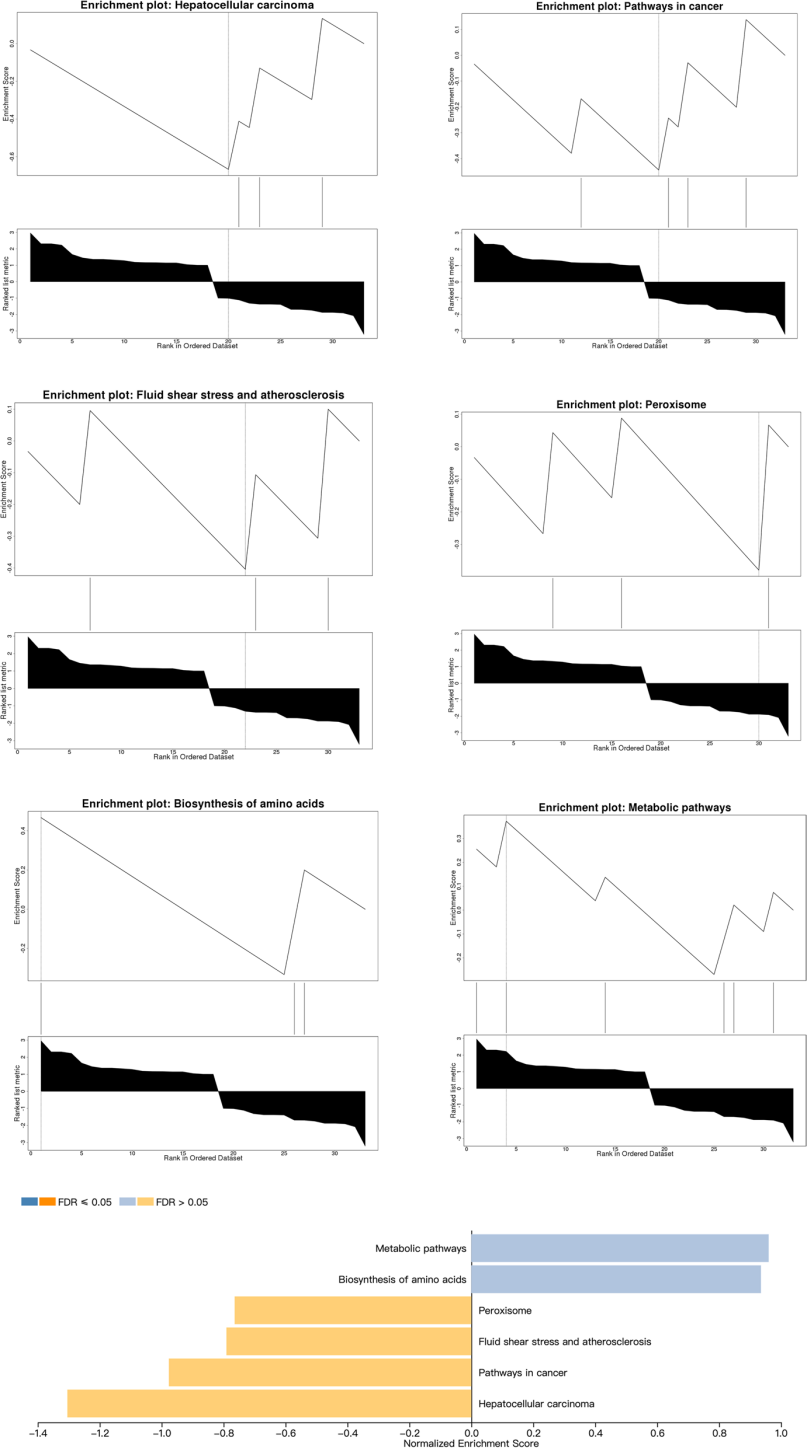
The GSEA results indicated that the significantly enriched genes were involved in the metabolic pathways, biosynthesis of amino acids peroxisome, fluid shear stress and atherosclerosis, pathway in cancer, and hepatocellular carcinoma

**Fig. 4:**
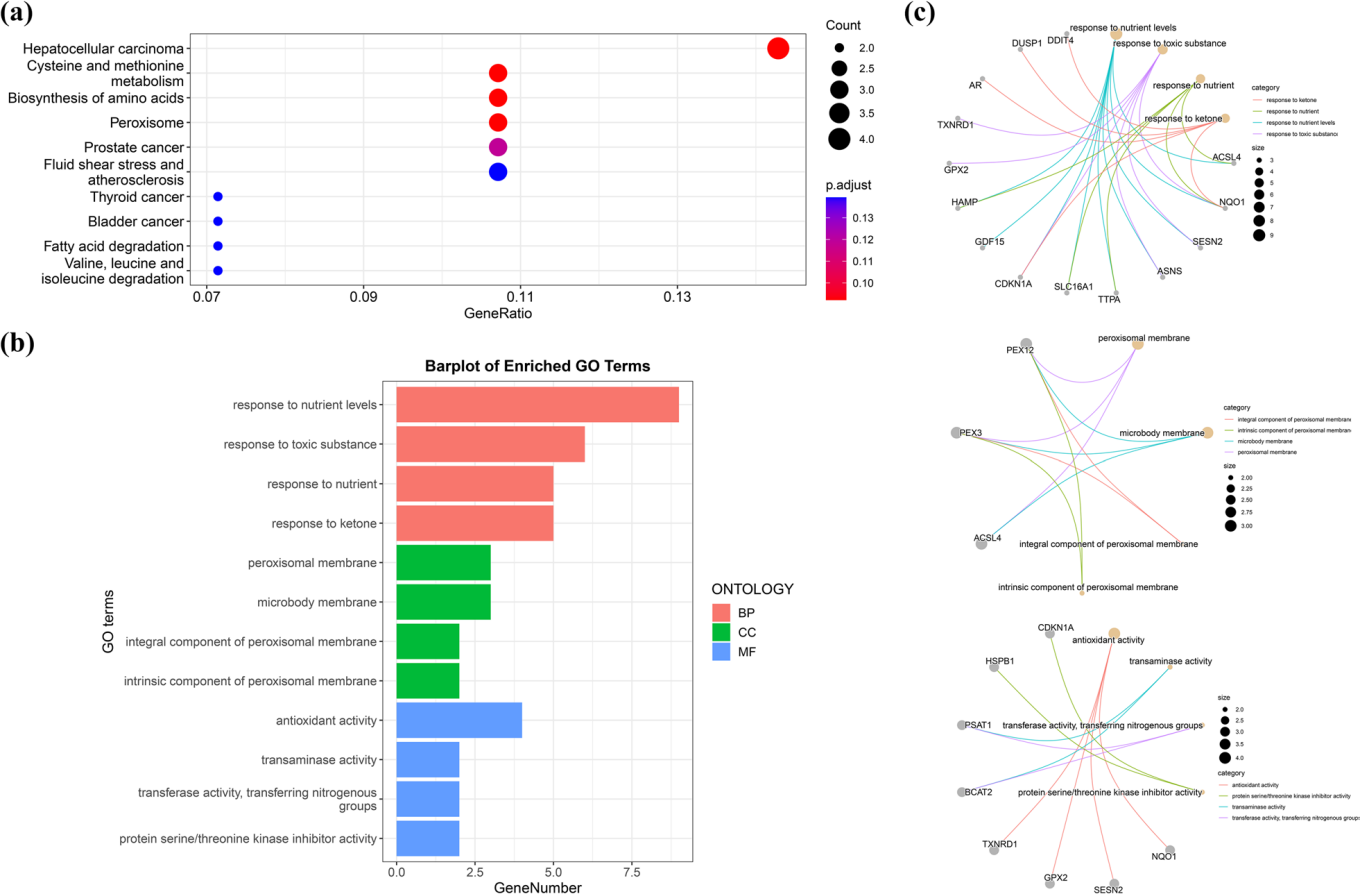
**A** Kyoto Encyclopedia of Genes and Genomes (KEGG) pathway analysis of ferroptosis-related DEGs. **B** Gene Ontology (GO) enrichment analysis of ferroptosis-related DEGs. **C** Network graph of GO enrichment of ferroptosis-related DEGs. The network graph shows the DEGs enriched in the top 4 GO BP, CC, and MF terms. The yellow points represent the GO categories, the color of the line delivered by a point indicates the category of the point in the legend, and the size of a point indicates the number of genes it includes. BPs: biological processes; CCs: cellular components; MFs: molecular functions

KEGG and GO enrichment analyses were performed on ferroptosis-related DEGs. The results of GSEA in WebGestalt software revealed that the genes were primarily enriched in metabolic pathways, biosynthesis of amino acids, peroxisome, fluid shear stress and atherosclerosis, pathway in cancer, and hepatocellular carcinoma (Fig. 3). KEGG enrichment analysis in DAVID indicated that cysteine and methionine metabolism, biosynthesis of amino acids, peroxisome, fluid shear stress and atherosclerosis, and biosynthesis of cofactors were significantly enriched in the gene sets (Table 4). The enrichKEGG function in the ClusterProfiler package was used to identify the pathways in which the DEGs were enriched. These DEGs were enriched in hepatocellular carcinoma, cysteine and methionine metabolism, biosynthesis of amino acids, peroxisome, prostate cancer, fluid shear stress and atherosclerosis (Fig. 4A). Notably, biosynthesis of amino acids, peroxisome, fluid shear stress and atherosclerosis were the main biological pathways involved and were identified by the WebGestalt, DAVID, and ClusterProfiler packages.

**Table 4:** KEGG enrichment analysis of DEGs by DAVID

Term	Count	%	*P* Value	Benjamimi	FDR
Cysteine and methionine metabolism	3	9.090909091	0.01101114	0.92536812	0.92536812
Biosynthesis of amino acids	3	9.090909091	0.02375332	0.92536812	0.92536812
Peroxisome	3	9.090909091	0.02804146	0.92536812	0.92536812
Fluid shear stress and atherosclerosis	3	9.090909091	0.07250731	1	1
Biosynthesis of cofactors	3	9.090909091	0.08556692	1	1

*FDR* false discovery rate

**Fig. 5:**
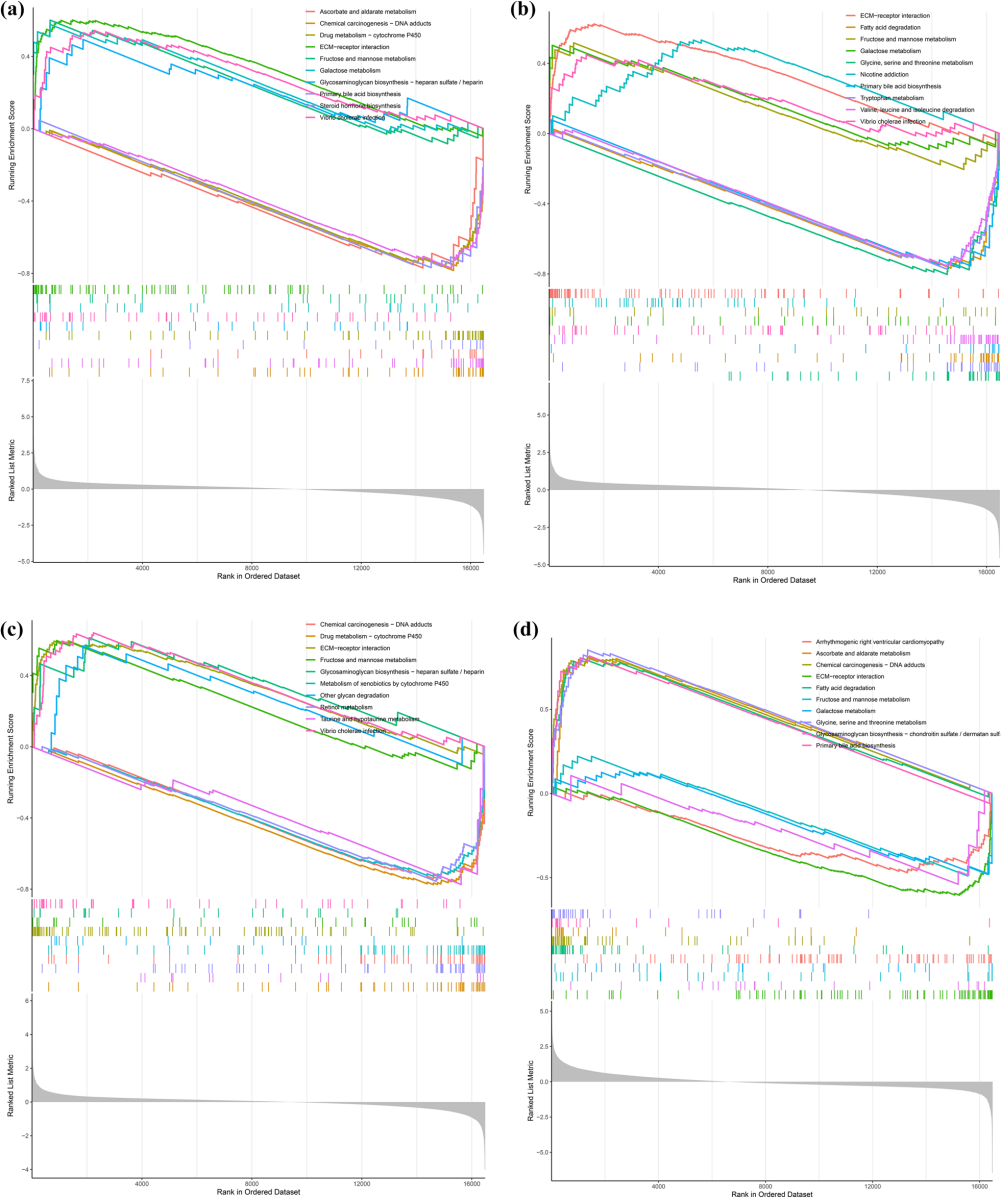

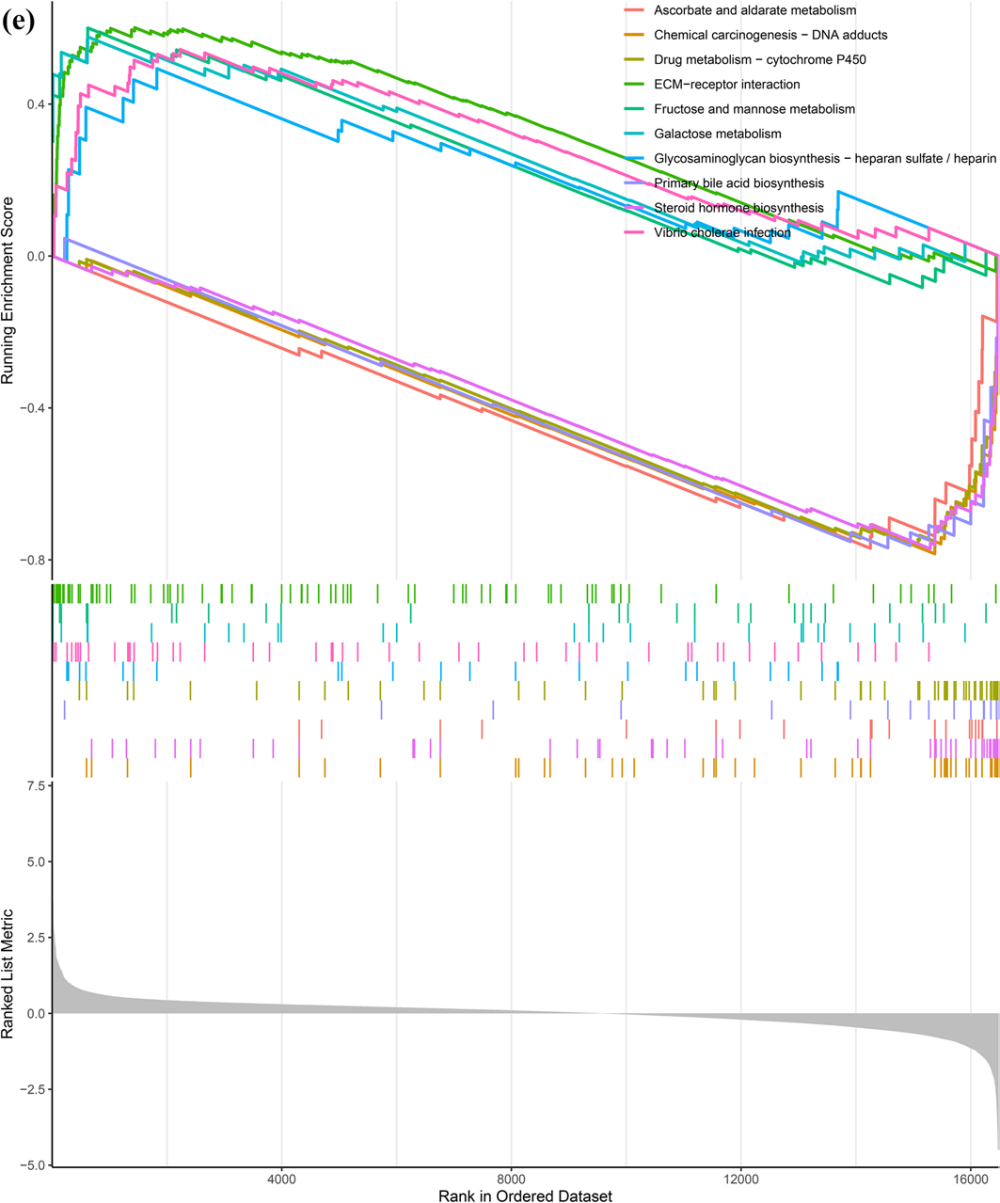
Gene set enrichment analysis (GSEA) revealed the enrichment pathways of the hub genes. **A** HRAS, **B** NQO1, **C** TXNRD1, **D** PSAT1, and **E** SQSTM1

In addition, the most significantly enriched terms in the GO analysis included BPs such as response to nutrient levels and response to toxic substance, CCs such as peroxisomal membrane and microbody membrane, and MFs such as antioxidant activity (Fig. 4B). The cnetplot function in the ClusterProfiler package was used to exhibit the genes enriched in the top 4 processes with the smallest P value of BPs, CCs, and MFs. Among them, ACSL4, NQO1, SESN2, TXNRD1, and CDKN1A were enriched in at least two BP, CC, and MF terms (Fig. 4C). GSEA of the 5 hub genes showed that they are mainly involved in the metabolic pathways of fructose and mannose metabolism, galactose metabolism, ascorbate and aldarate metabolism, drug metabolism-cytochrome P450 and the biosynthetic pathways of primary bile acid biosynthesis and glycosaminoglycan biosynthesis. They are also associated with ECM-receptor interactions, chemical carcinogenesis-DNA adducts, and Vibrio cholerae infection (Fig. 5).

### PPI network and hub genes of ferroptosis-related DEGs

**Fig. 6:**
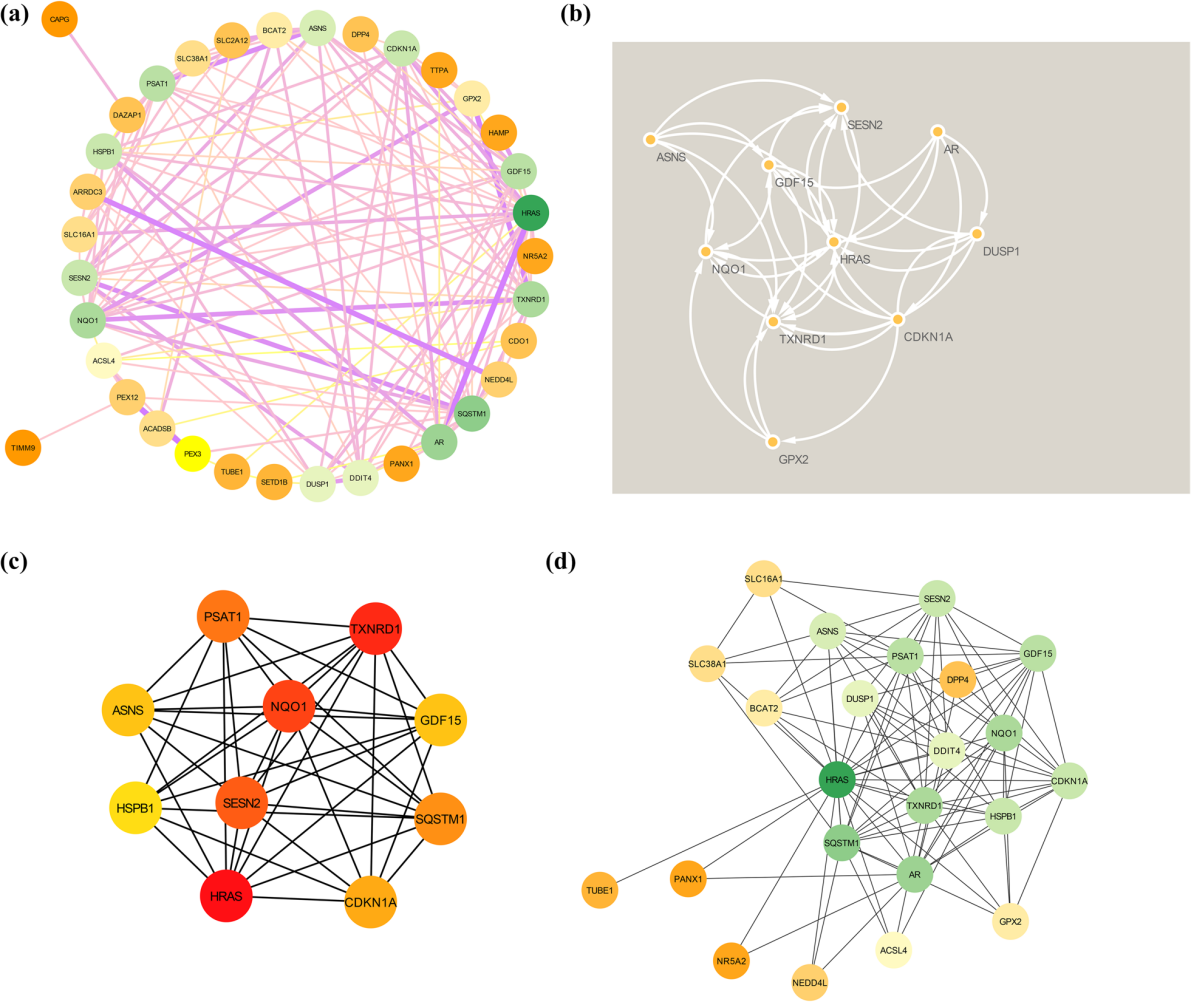
Construction of the PPI network of ferroptosis-related DEGs and screening of hub genes. **A** The STRING database was used to construct the PPI network of ferroptosis-related DEGs, with nodes and edges. The darker the color and the wider the edge, the stronger the evidence for the interaction between proteins. **B** The node gene cluster with the highest score constructed by the MCODE plug-in in Cytoscape consists of 10 genes. **C** cytoHubba was used to construct the top 10 hub genes. The figure shows the top 10 hub genes constructed by the MCC method. **D** cytoHubba was used to predict the first stop node genes that interact with HRAS. A total of 23 genes were predicted

We obtained a PPI network consisting of 35 nodes and 133 edges (Fig. 6A). MCODE in Cytoscape indicated that there is only one functional cluster with an established score > 5, including 10 genes and 34 edges (Fig. 6B). Five genes were screened in all 10 topological methods of CytoHubba in Cytoscape, namely, HRAS, TXNRD1, NQO1, PSAT1, and SQSTM1 (Additional file 2: Table S1, Fig. 6C). HRAS was the downregulated hub gene with the highest MCC and existed in the functional module. A total of 23 node genes were identified as interacting with HRAS by cytoHubba (Fig. 6D).

### Drugs identified in DrugBank

**Fig. 7:**
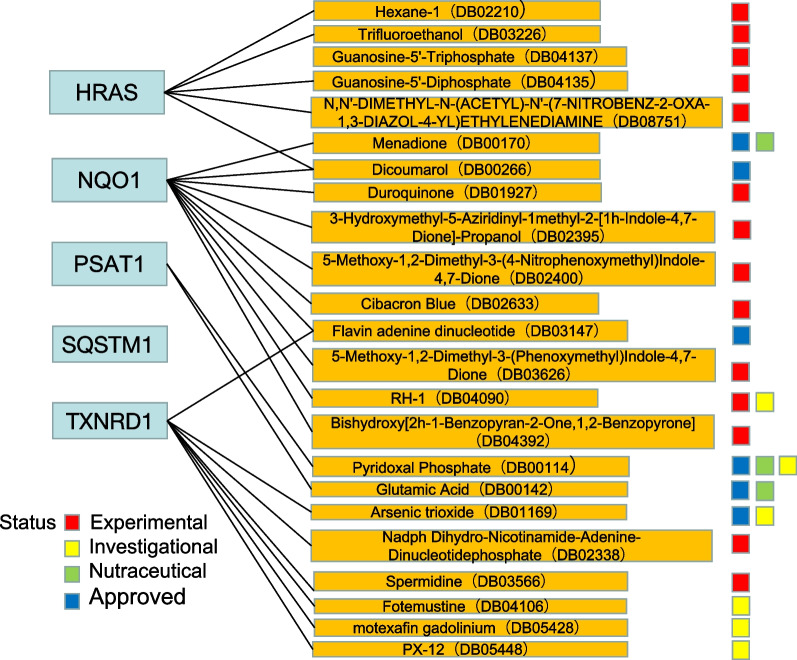
Drugs targeting these five hub genes were obtained from the DrugBank database. Drug status, including experimental, investigational, nutraceutical and approved, is indicated by colored squares

Based on drug and target information from the DrugBank database, 23 drugs targeting these five hub genes were identified (Fig. 7). No drugs targeting SQSTM1 have been identified. Among these drugs, six drugs are approved, including menadione (DB00170), DIC (DB00266), flavin adenine dinucleotide (DB03147), pyridoxal phosphate (DB00114), glutamic acid (DB00142), and arsenic trioxide (DB01169).

### Pathological morphology of rat liver tissue

**Fig. 8:**
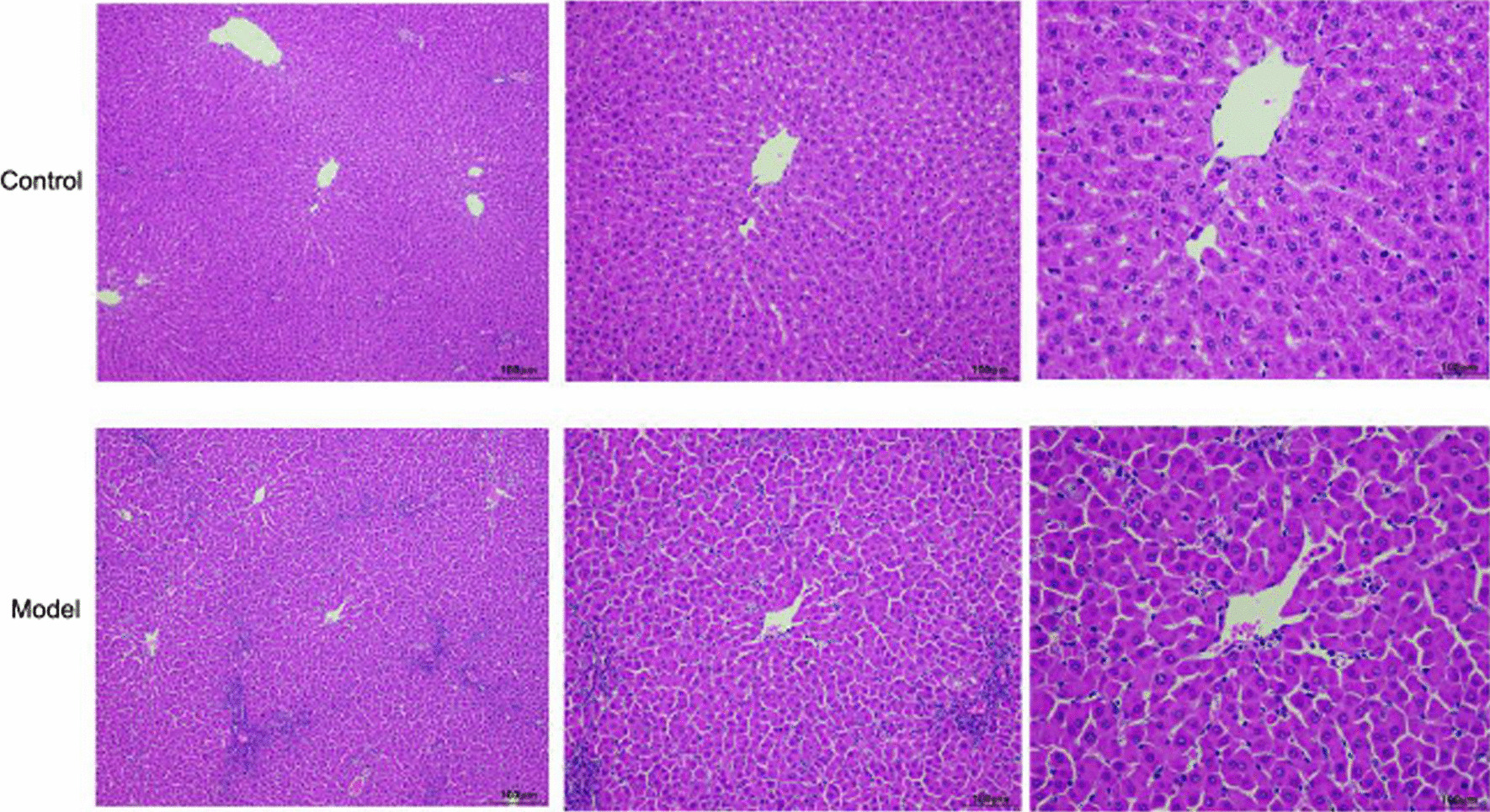
Pathological morphology of liver tissue of rats in the control and model groups (hematoxylin–eosin staining, × 100)

The pathological changes in the liver were observed by HE staining. The cells in the liver tissue of rats in the control group were arranged neatly, without denaturation or necrosis and no inflammatory cell infiltration. Cells in the ACLF group showed necrosis, inflammatory cell infiltration and fibrous tissue hyperplasia (Fig. 8).

### Potential biomarker expression by RT‒qPCR

**Fig. 9:**
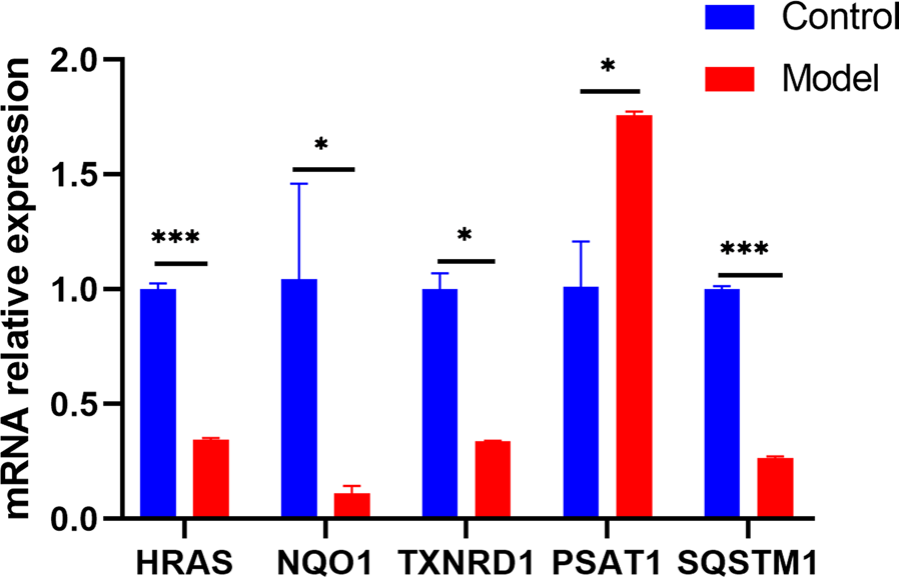
RT‒qPCR results showed that the expression levels of HRAS, TXNRD1, NQO1, and SQSTM1 were obviously lower and the expression level of PSAT1 was higher in ACLF rats than in healthy control rats. *P < 0.05, **P < 0.01, ***P < 0.001

Five hub genes, including HRAS, TXNRD1, NQO1, PSAT1, and SQSTM1, were validated through RT‒qPCR. The expression levels of HRAS, TXNRD1, NQO1, and SQSTM1 were clearly lower, while the expression level of PSAT1 was higher in the ACLF group than in the control group (Fig. 9).

## Discussion

In this study, we identified the hub genes related to ferroptosis and explored the potential role of ferroptosis in the pathogenesis of ACLF. Thirty-five DEGs were screened from the cross-comparison of GSE139602 and FerrDb datasets, including 16 downregulated genes and 19 upregulated genes. GO enrichment analysis suggested that the ferroptosis-related DEGs were mostly distributed in the peroxisomal membrane and microbody membrane and are involved in antioxidant activity and transaminase activity. The DEGs were suggested to mediate processes such as responses to nutrients and toxic substances. The GO enrichment network map demonstrated that ACSL4, NQO1, SESN2, TXNRD1, and CDKN1A were involved in multiple biological processes. These genes likely play a more critical role in ACLF. KEGG enrichment analysis indicated that these genes are mainly involved in the biosynthesis of amino acids pathways, peroxisomes, fluid shear stress and atherosclerosis pathways.

Ferroptosis is a newly discovered type of cell death characterized by lipid ROS accumulation due to intracellular iron overload [1]. Glutathione (GSH)/glutathione peroxidase (GPX4) is the classical regulatory pathway of ferroptosis [19]. Increasing evidence has shown that ferroptosis is one of the main triggers for the exacerbation of several liver diseases [5, 20–22]. The liver plays a vital role in iron metabolism, as it recirculates and stores iron, as well as synthesizes iron-containing enzymes. Iron overload can cause abnormalities in the mitochondrial oxidative phosphorylation pathway in hepatocytes, which can produce large amounts of ROS [23]. When the level of ROS exceeds the clearance level of the body's antioxidant system, it can oxidize unsaturated fatty acids on cell membranes and organelle membranes and directly or indirectly damage the structural function of hepatocytes, contributing to the progression of ferroptosis in liver cells [24].

The biosynthesis of amino acids is crucial for ferroptosis [25–29]. GSH is synthesized from three key amino acids, cysteine, glutamate, and glycine [30, 31]. Both glycine and cysteine can be produced through the metabolic conversion of serin from glucose. As an essential amino acid, serine is required not only for protein biosynthesis but also for the biosynthesis of numerous intracellular molecules, including GSH [32]. Sufficient cysteine levels contribute to the maintenance of redox homeostasis in cells and protects against oxidative death [22, 33]. Therefore, the metabolites and enzymes that convert serine and cysteine to GSH are essential to inhibit the occurrence of ferroptosis in liver cells.

HRAS, TXNRD1, NQO1, PSAT1, and SQSTM1 were simultaneously identified as the top hub genes. GSEA showed that the hub genes are mainly involved in the drug metabolism-cytochrome P450 metabolic pathway, the ECM-receptor interaction and glycosaminoglycan biosynthesis biosynthetic pathways, which are particularly closely related to ferroptosis. Cytochrome P450 oxidoreductase (POR) is an important component of the cytochrome P450 pathway that promotes ferroptosis by increasing the peroxidation of saturated phospholipids in the cell membrane [34, 35]. When cells are separated from the ECM, it can lead to a series of deleterious metabolic changes, including the triggering of ferritin deposition and the induction of ferroptosis [36]. Sulfated glycosaminoglycans (GAGs), especially chondroitin sulfate and heparan sulfate, have antioxidant effects [37, 38]. Sulfation plays an important role in maintaining cell survival and preventing oxidative stress-induced cell death [39].

Additionally, drugs targeting the above genes were retrieved from DrugBank, which consisted of six approved drugs. Flavin adenine dinucleotide (DB03147) is a coenzyme form of vitamin B2 used in clinical conditions associated with vitamin B2 deficiency, such as keratitis and blepharitis. Arsenic trioxide (DB01169) is a chemotherapeutic agent used in the treatment of refractory or relapsed acute promyelocytic leukemia in patients with prior retinoid and anthracycline chemotherapy. It is suspected that arsenic trisulfide induces cancer cells to undergo apoptosis. The enzyme thioredoxin reductase has recently been identified as a target for arsenic trioxide. Pyridoxal phosphate (DB00114) is the active form of vitamin B6, which serves as a coenzyme for the synthesis of amino acids and aminolevulinic acid. Dicoumarol (DB00266) is a coumarin-like compound found in sweet clover. It is used as an oral anticoagulant and acts by inhibiting the hepatic synthesis of vitamin K-dependent coagulation factors [40–42]. Menadione (DB00170), known as vitamin K3, is involved as a cofactor in the posttranslational gamma-carboxylation of glutamic acid residues of certain proteins in the body. These proteins include vitamin K-dependent coagulation factors II (prothrombin), VII (proconvertin), and a growth-arrest-specific factor (Gas6). Glutamic acid (DB00142) is an amino acid commonly found as a component in total parenteral nutrition and is one of the building blocks in protein synthesis [43]. In summary, the above drugs are mostly related to amino acids or vitamins, which may mediate the biosynthesis of the amino acids in ACLF. This finding provides a reference for identifying potential novel treatments against ferroptosis in ACLF.

The RT‒qPCR results showed that the expression levels of HRAS, TXNRD1, NQO1, and SQSTM1 were clearly lower, while the expression level of PSAT1 was higher in the ACLF group than in the control group, confirming the reliability of the bioinformatics analysis. Among them, PSAT1, TXNRD1, and HRAS expression affect cellular amino acid biosynthesis. PSAT1 is a critical regulator in the serine synthesis pathway (SSP). A study found that PSAT1, a member of the class-V family of pyridyl aldehyde phosphate-dependent aminotransferases, catalyzes the second step of SSP [44]. Specifically, a significant upregulation in the expression of key enzymes such as PSAT1 increased the levels of serine and its downstream products [45]. PSAT1 expression increased the level of GSH in cells through SSP, which increased oxidative stress tolerance and thus inhibited the occurrence of ferroptosis [46]. However, our results showed that the PSAT1 gene was highly expressed in the ACLF group, which is inconsistent with previous studies. In this regard, we speculate that the upregulation of PSAT1 expression may be the result of negative feedback of decreased PSAT1 enzyme activity in ACLF model rats. The decrease in PSAT1 enzyme activity may lead to negative feedback of cells to upregulate the expression of its coding gene PSAT1, which is conducive to maintaining the PSAT1 enzyme activity level. However, this process does not change the iron-related cell death caused by the decrease in enzyme activity, which can be considered resistance to ferroptosis. Thus, we will conduct future experimental studies and data analysis to investigate this hypothesis. Simultaneously, TXNRD1 is a critical regulator that may be involved in cysteine depletion-induced cellular ferroptosis. The TXNRD1 gene encodes thioredoxin reductase 1, a key antioxidant selenoprotease that regulates cellular redox homeostasis [47]. Some reports have suggested that the thioredoxin system regulates the dithiol/disulfide bond balance in proteins through disulfide bond reductase activity and protects against oxidative stress [48–50]. A previous study indicated that TXNRD1 was obviously upregulated in chronic myeloid leukemia cells after cysteine depletion [51]. Thioredoxin is also a special antioxidant that compensates for reduced GSH levels, reacts with ROS and is eventually recovered by TXNRD [52]. Cysteine is a limiting amino acid for glutathione synthesis and plays an irreplaceable role in maintaining redox balance [53]. Subsequently, ferroptosis occurred upon treatment with a TXNRD1 inhibitor after cysteine depletion. Therefore, it is reasonable to believe that when the gene expression of TXNRD1 is downregulated or the activity of TXNRD1 is inhibited, cysteine is depleted, eventually leading to ferroptosis. HRAS, referred to as oncogenic RAS with NRAS and KRAS, is one of the most frequently mutated driver proto-oncogenes in cancer. Oncogenic RAS abnormally rewires metabolic pathways, leading to ROS generation [54]. Then, ROS promote the accumulation of oxidative byproducts, decreasing the threshold for cancer cells to undergo ferroptosis. Moreover, oncogenic RAS also establishes ROS scavenging mechanisms to inhibit cellular senescence and promote tumor formation [55]. Oncogenic RAS promotes the transport of intracellular glutamate to the outside of the cell by affecting the system xc-transporter and simultaneously takes extracellular cystine into the cell and converts it into cysteine for the synthesis of GSH [56]. In summary, PSAT1 affects the serine synthesis pathway, TXNRD1 is involved in the cysteine depletion pathway, and HRAS affects the xc-transporter system to influence cysteine and glutamate synthesis, all three of which affect the synthesis of GSH, resulting in intracellular redox imbalance and promoting lipid peroxidation to generate ROS, thus causing ferroptosis.

**Fig. 10:**
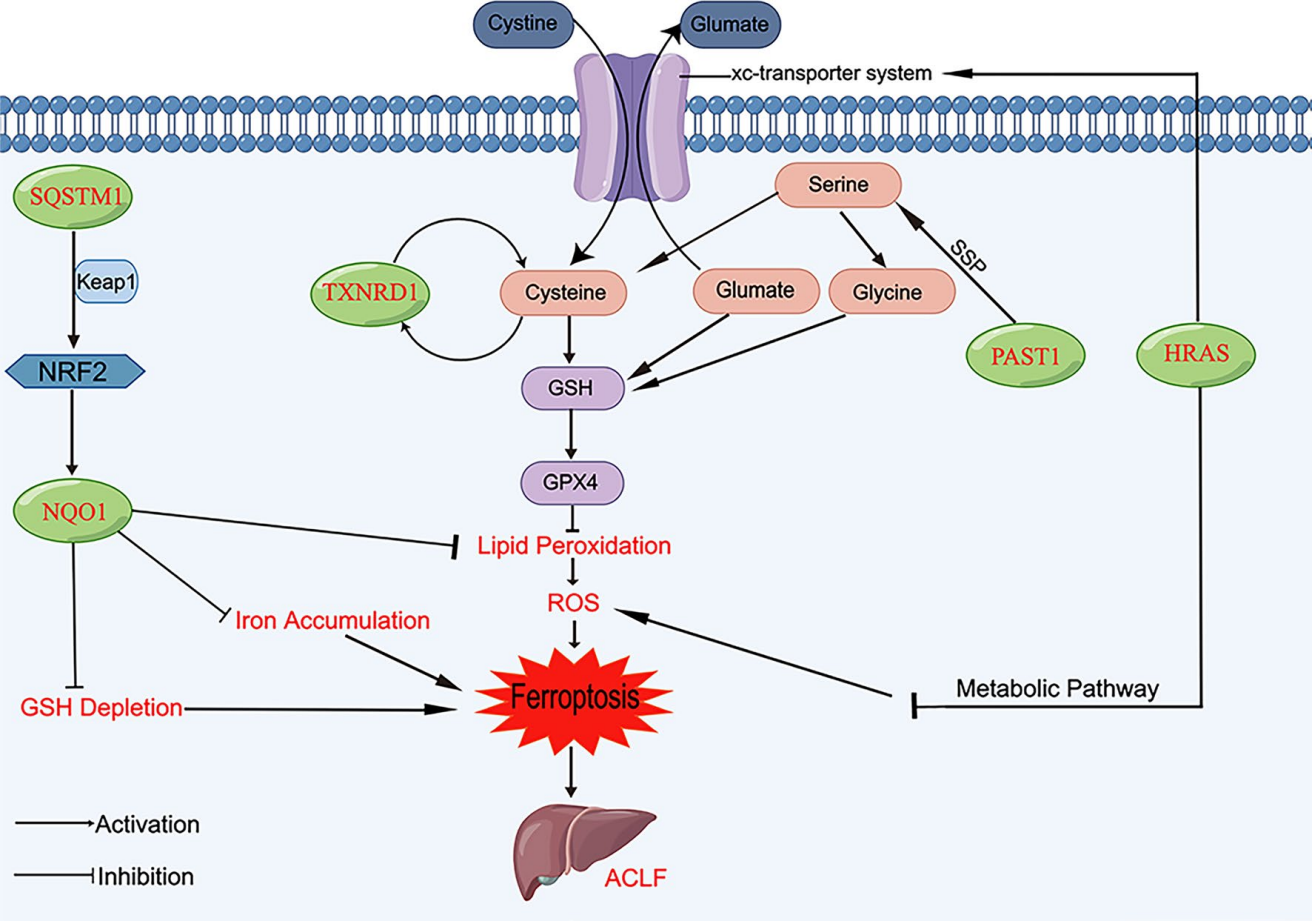
PSAT1 affects the serine synthesis pathway (SSP), TXNRD1 is involved in the cysteine depletion pathway, and HRAS indirectly inhibits ferroptosis through metabolic pathways and the xc-transporter. SQSTM1 may induce the Keap1/NRF2/NQO1 signaling pathway to protect against ferroptotic events in acute-on-chronic liver failure (ACLF)

In addition, the present study reveals that SQSTM1 and NQO1 may mediate ferroptosis by targeting the NRF2 pathway. SQSTM1, also known as p62, is an autophagy receptor [57]. A study by Sun et al. [58] provided the first evidence that activation of the p62-Keap1-NRF2 pathway protects hepatocellular carcinoma cells from ferroptosis. Irion metabolism and lipid peroxidation are modified by the NRF2-regulated genes NQO1, FTH1, and HO1, contributing to the inhibition of ferroptosis. Another study [59] also found that in the presence of high SQSTM1 expression, ferroptosis could be prevented by promoting the nuclear transfer of NRF2 and increasing heme oxygenase-1 expression. Knocking down SQSTM1 inhibited NRF2 expression and led to growth inhibition with increased ferroptotic events, including a reduction in GSH, an increase in lipid ROS levels, and an increase in iron levels. Daiha Shin’s study indicated that cell viability was reduced and cellular lipid ROS levels were increased when SQSTM1 was inhibited, which were reversed by a ferroptosis inducer (ferrostatin-1) [60]. NQO1 is a protective antioxidant agent and regulates oxidative stress in chromatin [61]. Interestingly, the NRF2-NQO1 axis represents a protective mechanism against ROS overproduction [62]. Thus, we infer that high SQSTM1 expression may activate the NRF2/NQO1 signaling pathway by binding to Keap1 against ferroptotic events, including lipid ROS production, GSH depletion, iron accumulation, and lipid peroxidation, thus protecting hepatocytes from ferroptosis. (Fig. 10).

There are some limitations to our study. The sample size in the GSE139602 dataset was small, which may lead to bias in the final gene validation results. The human liver tissue gene expression data are relatively few. In this regard, the mechanisms and specific signaling pathways of ferroptosis in ACLF can be further investigated by collecting data with a larger sample size. Moreover, there is a lack of validation of external datasets and clinical trials. To improve the prevention and treatment of ACLF, more ACLF human liver tissue expression profiles will be needed for further analysis.

## Conclusion

In conclusion, we explored the gene expression profiles of ACLF patient samples, identified 35 ferroptosis-related DEGs, and identified their participating biological processes and pathways. We also identified and validated 5 pivotal genes related to ferroptosis in ACLF, which are mainly involved in the amino acid biosynthesis pathway and NRF2 pathway. Targeting these genes and pathways in combination with a pharmacological inhibitor of ferroptosis may be a potential therapy for ACLF.

## Supplementary Information

**Additional file1**. **Figure S1**: The appearance of liver tissues in control and model groups.

**Additional file2**. **Table S1**: Top10 ferroptosis-related DEGs by 10 topological analysis methods of CytoHubba.

## Data Availability

The datasets used and analyzed during the current study are available from the corresponding author on reasonable request. NCBI Gene Expression Omnibus. https://www.ncbi.nlm.nih.gov/. Accessed 29 March 2022. Ferroptosis Database. http://www.datjar.com:40013/bt2104/. Accessed 3 April 2022. Venny2.1. https://bioinfogp.cnb.csic.es/tools/venny/index.html. Accessed 5 April 2022. WebGestalt database. http://www.webgestalt.org/. Accessed 9 April 2022. DAVID 6.8 database. https://david.ncifcrf.gov. Accessed 8 April 2022. STRING database. https://cn.string-db.org/. Accessed 20 April 2022. DrugBank database. https://go.drugbank.com/. Accessed 19 November 2022.

## Abbreviations

ACLF: Acute-on-chronic liver failure
ROS: Reactive oxygen species
APAP: Acetaminophen
ALF: Acute liver failure
DEGs: Differentially expressed genes
RT‒qPCR: Real-time quantitative PCR
GEO: Gene Expression Omnibus
FDR: False discovery rate
FC: Fold change
FerrDb: Ferroptosis database
GSEA: Gene Set Enrichment Analysis
KEGG: Kyoto Encyclopedia of Genes and Genomes
GO: Gene ontology
BPs: Biological processes
CCs: Cellular components
MFs: Molecular functions
PPI: Protein‒protein interaction
MCODE: Molecular complex detection
MCC: The most accurate
HE: Hematoxylin–eosin
LPS: Lipopolysaccharide
GSH: Glutathione
GPX4: Glutathione peroxidase 4
SSP: Serine synthesis pathway
