# Supplementary material for: Identification of potential ferroptosis hub genes in acute-on-chronic liver failure based on bioinformatics analysis and experimental verification
Source: BMC Med Genomics. 2023 Mar 11;16:52. doi: 10.1186/s12920-023-01480-4 (PMC10007765; doi:10.1186/s12920-023-01480-4)
Supplement: Supplementary file 1 — Additional file1. Figure S1: The appearance of liver tissues in control and model groups. [file 12920_2023_1480_MOESM1_ESM.docx]

Supplementary Figure 1. The appearance of liver tissues in control and model groups.
