# Supplementary material for: Identification of potential ferroptosis hub genes in acute-on-chronic liver failure based on bioinformatics analysis and experimental verification
Source: BMC Med Genomics. 2023 Mar 11;16:52. doi: 10.1186/s12920-023-01480-4 (PMC10007765; doi:10.1186/s12920-023-01480-4)
Supplement: Supplementary file 2 — Additional file2. Table S1: Top10 ferroptosis-related DEGs by 10 topological analysis methods of CytoHubba. [file 12920_2023_1480_MOESM2_ESM.docx]

Supplementary Table 1. ﻿Top10 ferroptosis-related DEGs by 10 topological analysis methods of CytoHubba.

| MCC | MNC | Degree | EPC | BottleNeck | EcCentricity | Closeness | Radiality | Betweenness | Stress |
| --- | --- | --- | --- | --- | --- | --- | --- | --- | --- |
| HRAS | HRAS | HRAS | HRAS | HRAS | ASNS | HRAS | HRAS | HRAS | HRAS |
| TXNRD1 | SQSTM1 | SQSTM1 | SQSTM1 | SQSTM1 | SLC38A1 | SQSTM1 | SQSTM1 | SQSTM1 | SQSTM1 |
| NQO1 | NQO1 | AR | TXNRD1 | NQO1 | SESN2 | NQO1 | NQO1 | AR | AR |
| SESN2 | TXNRD1 | NQO1 | NQO1 | AR | BCAT2 | AR | TXNRD1 | NQO1 | NQO1 |
| PSAT1 | AR | TXNRD1 | PSAT1 | PSAT1 | PSAT1 | TXNRD1 | AR | PEX12 | TXNRD1 |
| SQSTM1 | PSAT1 | PSAT1 | SESN2 | ACSL4 | ACSL4 | PSAT1 | PSAT1 | DAZAP1 | HSPB1 |
| CDKN1A | GDF15 | GDF15 | GDF15 | TXNRD1 | NQO1 | GDF15 | SESN2 | PSAT1 | ACSL4 |
| ASNS | SESN2 | SESN2 | CDKN1A | ASNS | TXNRD1 | HSPB1 | HSPB1 | TXNRD1 | PSAT1 |
| GDF15 | HSPB1 | HSPB1 | AR | TUBE1 | SQSTM1 | SESN2 | CDKN1A | HSPB1 | DAZAP1 |
| HSPB1 | CDKN1A | CDKN1A | HSPB1 | PEX12 | HRAS | CDKN1A | GDF15 | ACSL4 | ASNS |
